# Elevated humoral response to cytomegalovirus in HIV-infected individuals with poor CD4^+^ T-cell immune recovery

**DOI:** 10.1371/journal.pone.0184433

**Published:** 2017-09-21

**Authors:** Elisabet Gómez-Mora, Marta Massanella, Elisabet García, David Giles, Marta Bernadó, Victor Urrea, Jorge Carrillo, Dan Ouchi, Jordi Puig, Eugenia Negredo, Bonaventura Clotet, Julià Blanco, Cecilia Cabrera

**Affiliations:** 1 IrsiCaixa AIDS Research Institute, Institut de Recerca Germans Trias i Pujol (IGTP), Hospital Universitari Germans Trias i Pujol, Universitat Autonoma de Barcelona, Badalona, Barcelona, Spain; 2 Université de Montréal, Faculté de Médecine, Department of Microbiology, Infectiology and Immunology, Centre de Recherche du CHUM, Montréal, QC, Canada; 3 Biokit, Lliçà d’Amunt, Barcelona, Spain; 4 Fundació Lluita contra la SIDA, Hospital Universitari Germans Trias i Pujol, Badalona, Barcelona, Spain; 5 Universitat de Vic-Central de Catalunya, UVIC-UCC, Vic, Spain; University of San Francisco, UNITED STATES

## Abstract

Some HIV-infected c-ART-suppressed individuals show incomplete CD4^+^ T-cell recovery, abnormal T-cell activation and higher mortality. One potential source of immune activation could be coinfection with cytomegalovirus (CMV). IgG and IgM levels, immune activation, inflammation and T-cell death in c-ART-suppressed individuals with CD4^+^ T-cell counts >350 cells/μL (immunoconcordant, n = 133) or <350 cells/μL (immunodiscordant, n = 95) were analyzed to evaluate the effect of CMV humoral response on immune recovery. In total, 27 HIV-uninfected individuals were included as controls. In addition, the presence of CMV IgM antibodies was retrospectively analyzed in 58 immunoconcordant individuals and 66 immunodiscordant individuals. Increased CMV IgG levels were observed in individuals with poor immune reconstitution (p = 0.0002). Increased CMV IgG responses were significantly correlated with lower nadir and absolute CD4^+^ T-cell counts. In contrast, CMV IgG responses were positively correlated with activation (HLA-DR^+^) and death markers in CD4^+^ T-cells and activated memory CD8^+^ T-cells (CD45RA^-^CD38^+^). Longitudinal subanalysis revealed an increased frequency of IgM^+^ samples in individuals with poor CD4^+^ T-cell recovery, and an association was observed between retrospective IgM positivity and the current level of IgG. The magnitude of the humoral immune response to CMV is associated with nadir CD4^+^ T-cell counts, inflammation, immune activation and CD4^+^ T-cell death, thus suggesting that CMV infection may be a relevant driving force in the increased morbidity/mortality observed in HIV^+^ individuals with poor CD4^+^ T-cell recovery.

## Introduction

Combination antiretroviral therapy (c-ART) has dramatically improved the health of HIV-infected individuals. However, even for those HIV-infected individuals who are successfully treated, life expectancy remains reduced, particularly for individuals who fail to recover CD4^+^ T-cell counts [1]. These individuals, called immunodiscordants or immune non-responders, show higher levels of immune activation, inflammation, and immunosenescence, and they have a higher risk of AIDS-related and non-AIDS-related mortality and morbidity than immunoconcordant individuals, i.e., those who successfully recover normal CD4^+^ T-cell counts. The reason for this suboptimal immune reconstitution is not completely understood, and among other factors, persistent coinfections with other pathogens, including cytomegalovirus (CMV), are likely contributing factors (reviewed in [2]).

CMV is a highly prevalent beta herpesvirus that after infection establishes lifelong latency within the host and periodically reactivates when the cellular immune system is compromised in response to inflammation, infection or stress [3,4]. IgM antibodies are the first antibodies produced after a primary infection. IgM levels increase for a short time after infection and decrease to below detectable levels after 2–3 months [5]. The presence of IgM antibodies, however, cannot be exclusively used to diagnose primary CMV infection, because they are also produced during viral reactivation or reinfection with a different CMV strain [5–7]. IgG antibodies are produced several weeks after the initial CMV infection. IgG levels increase during active infection and then stabilize as the CMV infection resolves and becomes inactive. Two to four months after infection, these IgG antibodies mature from low to high avidity (high binding strength) [8–10]. Therefore, measurement of CMV IgG avidity has emerged as the “gold standard” for distinguishing primary (IgM^+^ and low-avidity IgG) from non-primary CMV infection (IgM^+^ and high-avidity IgG) and is being used worldwide to identify primary CMV infection during pregnancy [7,10,11].

In HIV-uninfected individuals, CMV IgG seropositivity has been clearly associated with immunosenescence and the development of cardiovascular diseases, cancer and all-cause mortality [12,13]. In HIV-infected individuals, coinfection has been implicated in immune activation, senescence, cardiovascular complications and accelerated progression to AIDS and death [14–16]. The humoral immune response to CMV, as measured by circulating anti-CMV IgG antibodies, has been associated with disease progression [17–19], risk of non-AIDS related events [15] cardiovascular disease [20], impaired neurocognitive function [21] and physical function impairment [22]. Similarly, in HIV-uninfected individuals, different studies have also shown that CMV-specific humoral immune responses were associated with cardiovascular disease and all-cause mortality [12,23–25]. These observations suggest that the magnitude of the humoral immune response may be a relevant marker of the deleterious effects of CMV infection.

Therefore, in this study, we aimed to investigate the association between humoral response, CMV IgG and IgM levels and CD4 immune recovery, immune activation and cell death in long-term cART-suppressed HIV-infected individuals with favorable and unfavorable immunologic responses. Our results suggest that in long-term-treated HIV-infected individuals with poor CD4^+^T-cell recovery, subclinical CMV reactivation appears to be more frequent and to be associated with increased CMV humoral response, CD4^+^ T-cell activation and cell death.

## Methods

### Study population and samples

Individuals on long-term c-ART (median 10 years) with viral load <50 copies/mL (n = 228) were classified according to their CD4^+^ T-cell counts as previously described [26,27]: individuals with adequate immune recovery, referred as immunoconcordant, with absolute CD4^+^ T-cell counts >350 cells/μL (n = 133), or individuals with poor immune reconstitution, referred as immunodiscordant, with absolute CD4^+^ T-cell counts <350 cells/μL (n = 95). In addition, a group of HIV-uninfected individuals (HIV^-^) were selected as the control group (Table 1). The institutional review board on biomedical research from Hospital Germans Trias i Pujol approved this study (EO code: EO-07-024). The methods were carried out in accordance with the Declaration of Helsinki. Written informed consent was obtained from all participants.

**Table 1:** Participant characteristics.

				HIV^+^ subgroups		
	HIV^-^ (n = 27)	All HIV^+^ (n = 228)	*p* value^a^	Immunoconcordant (n = 133)	Immunodiscordant (n = 95)	*p* value^b^
Age (years); median (IQR)	38 (33–50)	45 (41–50)	***0*.*0147***	44 (40–50)	46 (43–50)	***0*.*0464***
Gender (males); n. (%)	11 (40.7)	176 (77.2)	***0*.*0002***^***c***^	99 (74.4)	77 (81)	*0*.*2655*^*c*^
Time since HIV diagnosis (years); median (IQR)	NA	12.87 (8.2–17.4)		12.82 (9.2–16.4)	13.71 (7–19.9)	*0*.*4957*
Time on ART (years); median (IQR)	NA	10.62 (6.7–13.4)		11.08 (7.8–13.3)	9.27 (4.4–14.4)	*0*.*1058*
Nadir CD4 T-cell counts (cells/μL); median (IQR)	NA	142.5 (63.3–263)		234 (129.5–318)	71 (28–135)	***< 0*.*0001***
Absolute CD4 T-cell count (cells/μL); median (IQR)	782 (491–1012)	443 (285–674)	***0*.*0001***	632 (480.5–797)	250.5 (201.5–315.5)	***< 0*.*0001***
% CD4 T-cell; median (IQR)	59 (53–62)	26 (19–33)	***<0*.*0001***	30 (26–36)	18 (14–21)	***< 0*.*0001***
Absolute CD8 T-cell count (cells/μL); median (IQR)	453 (353–582)	783 (601–1067)	***<0*.*0001***	809 (672–1115)	705 (508–958)	*0*.*006*
% CD8 T-cell; median (IQR)	35 (30–39)	46 (38–52)	***<0*.*0001***	41 (35–48)	51 (45–58)	***< 0*.*0001***
CMV coinfection, no. (%)	17(63)	213 (93)	***<0*.*0001***^***c***^	124 (93)	89 (94)	*1*.*0*^*c*^
HCV coinfection, n. (%)	ND			43 (32.3)	43 (45.3)	*0*.*0531*^*c*^
HBV coinfection, n. (%)	ND			6 (4.5)	4 (4.2)	*1*.*0*^*c*^

^a^Comparison of HIV- and HIV+ individuals.

^b^Comparison of immunoconcordant and immunodiscordant individuals. (Mann Whitney U test or ^c^Fisher’s exact test). NA: Not Applicable, ND: Not determined.

A single blood sample was drawn from each participant. Blood was immediately stained and processed. Plasma was obtained by centrifugation of blood at 1200 g for 10 minutes and was stored at -80°C. All plasma samples were screened for CMV IgG and IgM antibodies. Peripheral blood mononuclear cells (PBMCs) were obtained from cell concentrates layered on Ficoll–Hypaque density gradients (Atom Reactiva) and were used immediately for *ex vivo* cell death assays. For the retrospective longitudinal analysis, available stored plasma samples from immunoconcordant and immunodiscordant individuals were assayed for the presence of CMV IgM.

### Anti-CMV IgG and IgM antibody levels and IgG avidity

The CMV-specific IgG and IgM antibodies were measured using the commercial semi-quantitative BIO-FLASH® CMV IgG and the qualitative BIO-FLASH® CMV IgM chemiluminescent immunoassays, respectively (Biokit, Barcelona, Spain) according to the manufacturer’s instructions. Briefly, BIO-FLASH CMV IgG or IgM paramagnetic microparticles (coated with CMV antigens) were mixed and incubated with the sample. Magnetic separation followed by a wash step was performed to remove residual sample. Then, a tracer consisting of an isoluminol-labeled monoclonal antibody anti-human IgG or IgM was immediately added. After a second incubation, magnetic separation and washing, reagents triggering the chemiluminescent reaction were added. The emitted light was measured in relative light units (RLU) by the BIO-FLASH luminometer (Biokit, Barcelona, Spain). The amount of anti-CMV IgG or anti-CMV IgM in each sample was determined from the measured emitted light (RLU) by interpolation in a working calibration curve generated by the use of calibrators. The BIO-FLASH CMV IgG calibrators were standardized over multiple runs on the BIO-FLASH instrument by using specific lots of reagents and against internal standards. Because no international standard is available, results are expressed in AU/mL. The BIO-FLASH CMV IgM results are reported in Signal/Cut-Off (S/CO). IgG avidity was determined by LABCO using a commercially available CMV IgG avidity kit based on an enzyme immunoassay method (ELISA) that employs urea (anti-CMV IgG Avidity ELISA, DRG, New Jersey, USA). Antigen-bound low-avidity IgG but not high-avidity IgG dissociates from the antigen in the presence of mild protein denaturants, such as urea. The avidity index was calculated as a proportion of the optical densities in the presence of urea and without urea. CMV IgG antibodies with avidities of >40% were considered high-avidity antibodies.

### Detection of CMV DNA by quantitative TaqMan PCR

DNA extraction was performed on 200 μl of plasma using a QIAamp DNA blood kit (Qiagen, Spain). The real-time polymerase chain reaction amplification for CMV detection (REALQUALITY RQ-CMV, AB Analitica, Italy) was used according to the manufacturer’s instructions.

### Immune activation, T-cell production/destruction and soluble CD14 assessments

The frequency of CD4^+^ and CD8^+^ T-cell subsets of immune activation and thymic production was analyzed in blood samples by flow cytometry using the following antibody panels: 1) CD95-FITC, PD-1-PE and HLA-DR-PerCP and 2) CD45RA-FITC, CD31-PE and CD38-PerCP with the common backbone in both tubes of CD3-APC-Cy7, CD4-APC and CD8-PE-Cy7 as previously reported [26]. Briefly, 20 μL of fresh blood was incubated (15 minutes at room temperature) with the different antibody combinations and lysed with 200 μL of FACS lysing solution (Becton Dickinson) for 30 minutes at room temperature. Cells were washed in PBS and resuspended in PBS 1% formaldehyde, acquired in a in a LSRII flow cytometer (Becton Dickinson) and analyzed with FlowJo software (Tree Star). An unstained control and a control antibody combination containing CD3–APC–Cy7, CD4–APC and CD8–PE–Cy7 were performed for all samples. The gating strategy is shown in S1 Fig. Cell death was evaluated as previously described [26,27]. Briefly, fresh PBMCs (200,000 cells) were cultivated in 96-well plates in 100 μL of RPMI medium supplemented with 10% FCS (fetal calf serum) for 24 hours in the absence or presence of the pancaspase inhibitor Z-VAD-fmk (R & D Systems). Cells were analyzed in a LSRII flow cytometer (Becton Dickinson) after incubation with 40 nM of the potentiometric mitochondrial probe DiOC_6_ (Invitrogen), 5 mg/mL propidium iodide (Sigma), and CD3- APC-Cy7, CD4-APC, and CD8-PE-Cy7 antibodies. Total cell death was calculated as the percentage of cells that showed low DiOC_6_ staining in control cultures. We performed additional analyses of necrotic cell death (caspase-independent), which was defined as the percentage of propidium iodide–stained cells in cultures that contained Z-VAD-fmk. Apoptotic cell death was defined as caspase-dependent death and was calculated by subtracting necrosis from total cell death. Intrinsic apoptosis was defined as the percentage of cells that showed low DiOC_6_ staining and that remained negative for propidium iodide in the presence of Z-VAD-fmk. Extrinsic apoptosis was calculated as the difference between total and intrinsic apoptosis (Gating strategy S2 Fig). Soluble CD14 (sCD14) levels in plasma were quantified by a commercially available ELISA (Diaclone) in stored plasma samples following manufacturer’s instructions,

### Statistical analysis

Continuous variables were expressed as the median with interquartile ranges and compared using nonparametric tests (two-tailed Mann–Whitney *U*-test) because the parameters were not normally distributed. For comparisons between groups p-values have been corrected for multiple testing using Holm’s method. Discrete variables were described as percentages and analyzed using the chi-square or Fisher’s exact test. Spearman’s correlation coefficient was calculated to identify associations between variables. The calculated p-values in the correlation analysis were corrected for multiple testing by using the false discovery rate (FDR) method. The relationship between reactivation rates and HIV subgroups restricted to individuals with reactivations was assessed by fitting a Poisson regression with the number of reactivations as the dependent variable and the HIV group indicator as the predictor. To normalize by the number of tested samples per patient, the logarithm of the number of samples was added as an offset variable in the model. Significance was analyzed through inference on the predictor’s coefficient (Wald test). All analyses and graphical representations were completed in GraphPad Prism v5.0a (GraphPad Software, Inc) or R 3.3.1 (R Core Team) [28].

## Results

### Participant characteristics

Table 1 summarizes the main characteristics of individuals enrolled in this study. A total of 228 HIV^+^ individuals were included: 133 were defined as immunoconcordants (individuals with favorable immunologic response) and 95 as immunodiscordants (unfavorable immunologic response). A minimal but significant difference was observed in the age, with immunodiscordant individuals being slightly older (median age, 46) than immunoconcordants (median age, 44, p = *0*.*046*). As previously reported, significantly lower CD4^+^ T-cell counts (absolute and percentage) determined at the time of recruitment and CD4 nadir (the lowest recorded value of the CD4 count) were observed in immunodiscordant subjects compared with immunoconcordant subjects [26]. In HIV^+^ individuals, CMV seropositivity was significantly higher than that in HIV-uninfected individuals (93% and 63%, respectively; p<0.0001). No difference in CMV seropositivity was found between HIV^+^ subgroups (Table 1). The prevalence of HCV and HBV did not significantly differ among immunodiscordants and immunoconcordants. Twenty-seven HIV-uninfected individuals were also included as a control group. This HIV^-^ group was younger and contained a lower proportion of males than the HIV^+^ group.

### Association between CMV IgG antibody levels and HIV status

**Fig 1:**
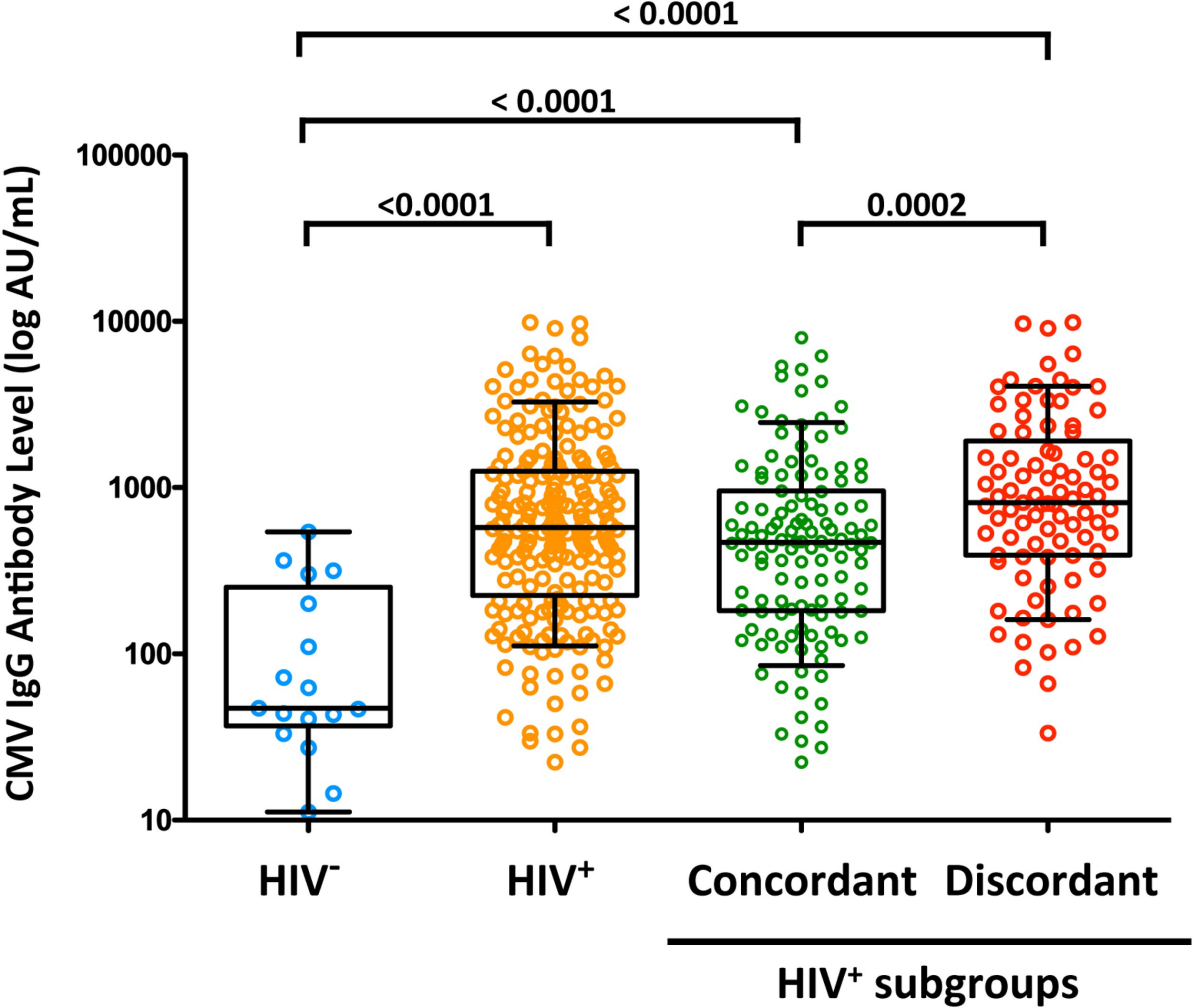
CMV IgG levels in the study participants. The IgG level was determined in plasma samples from healthy HIV-uninfected individuals (blue dots), HIV-positive individuals (orange dots), immunoconcordant individuals (green dots) and immunodiscordant individuals (red dots). The boxes represent the median and interquartile range of the values. Individual data of all subjects is displayed. The median values were compared using a non-parametric Mann-Whitney *U*-test. P-values have been corrected for multiple testing using Holm’s method.

The plasma CMV IgG antibody levels ranged from <10 to 9,850 AU/ml, including 25 participants (10 HIV^−^and 15 HIV^+^) with concentrations below the manufacturer’s cutoff for positive results (10 AU/ml). These IgG^-^ individuals were excluded from the subsequent analysis. CMV IgG level was not associated with age in HIV-uninfected individuals (p = 0.52) or in HIV^+^ individuals (p = 0.35. Table 2). Compared with HIV-uninfected controls, HIV-infected subjects had significantly higher plasma CMV IgG levels (p<0.0001) with a significantly higher level in all comparable age groups (Fig 1 and Table 3). Among HIV-infected individuals, significantly higher CMV IgG levels were detected in immunodiscordant subjects compared with immunoconcordant subjects (Fig 1), with significantly higher levels in all age groups analyzed (Table 3). No significant differences in the IgG levels were found between men and women in any of subgroups evaluated (data not shown).

**Table 2:** Associations of CMV IgG with sociodemographic, clinical and immunological variables.

Variable	r^a^	*p*-value	Adjusted p-value^b^
Age (years)	0.0744	0.2797	*0*.*3543*
Time since HIV diagnosis (years)	-0.0145	0.8328	*0*.*8394*
Time on ART (years)	-0.0166	0.8092	*0*.*8394*
Nadir CD4^+^ T-cell counts (cells/μL)	-0.2888	**< 0.0001**	***< 0*.*001***
Absolute CD4^+^ T-cell count (cells/μL)	-0.2941	**< 0.0001**	***< 0*.*001***
% CD4^+^ T-cells	-0.2836	**< 0.0001**	***< 0*.*001***
Absolute CD8 T-cell count (cells/μL)	0.1010	0.1429	*0*.*2334*
% CD8^+^ T-cells	0.3055	**< 0.0001**	***< 0*.*001***
% CD4^+^CD45RA^+^	-0.0977	0.1561	*0*.*2373*
% CD8^+^CD45RA^+^	-0.1561	**0.0230**	*0*.*0546*
**Activation**			
CD14 soluble (ng/ml)	0.1539	**0.0251**	*0*.*0561*
**CD4**^+^ **T cells**			
% CD4^+^CD38^+^	0.0262	0.7035	*0*.*7638*
% CD4^+^CD38^+^ CD45RA^+^	-0.1034	0.1336	*0*.*2308*
% CD4^+^CD38^+^ CD45RA^-^	0.1224	0.0755	*0*.*1434*
% CD4^+^HLADR^+^	0.1725	**0.0121**	***0*.*0328***
**CD8**^**+**^ **T cells**			
% CD8^+^CD38^+^	0.0960	0.1635	*0*.*2376*
% CD8^+^CD38^+^ CD45RA^+^	-0.0660	0.3466	*0*.*4249*
% CD8^+^CD38^+^ CD45RA^-^	0.1890	**0.0058**	***0*.*0184***
% CD8^+^HLADR^+^	0.0792	0.2520	*0*.*3302*
**T-cell production and destruction**			
**CD4**^**+**^ **T cells**			
% CD4^+^CD45RA^+^CD31^+^	-0.1187	0.0846	*0*.*1531*
% CD4^+^CD95^+^	0.2441	**0.0003**	***0*.*0023***
% CD4^+^HLADR^+^CD95^+^	0.2263	**0.0009**	***0*.*0049***
% CD4^+^CD95^+^PD1^+^	0.1965	**0.0042**	***0*.*0145***
% CD4^+^PD1^+^	0.1254	0.0692	*0*.*1384*
% CD4^+^HLADR^+^PD1^+^	0.1828	**0.0078**	***0*.*0228***
% CD4^+^ Total Death	0.2366	**0.0007**	***0*.*0044***
% CD4^+^ Necrosis	0.2299	**0.0011**	***0*.*0052***
% CD4^+^ Apoptosis	0.2085	**0.0030**	***0*.*0114***
% CD4^+^ Intrinsic Apoptosis	0.2260	**0.0013**	***0*.*0055***
% CD4^+^ Extrinsic Apoptosis	0.1416	**0.0455**	*0*.*0961*
**CD8**^**+**^ **T cells**			
% CD8^+^CD45RA^+^CD31^+^	-0.0941	0.1718	*0*.*2376*
% CD8^+^CD95^+^	0.1640	**0.0171**	***0*.*0433***
% CD8^+^HLADR^+^CD95^+^	0.0937	0.1751	*0*.*2376*
% CD8^+^ Total Death	0.0399	0.5745	*0*.*6615*
% CD8^+^ Necrosis	0.1028	0.1474	*0*.*2334*
% CD8^+^ Apoptosis	0.0144	0.8394	*0*.*8394*
% CD8^+^ Intrinsic Apoptosis	0.0366	0.6059	*0*.*6772*
% CD8+ Extrinsic Apoptosis	0.05913	0.4317	*0*.*5126*

^**a**^r represent Spearman correlations.

^**b**^FDR-adjusted p-values

**Table 3:** CMV IgG levels (values expressed in arbitrary units (AU)/mL).

				HIV^+^ subgroups		
Age (yr)	HIV^-^	All HIV^+^	*p-* value^a^	Immunoconcordant	Immunodiscordant	*p-* value^b^
**25–39**	63 (47–200)	480 (148–792)	***0*.*0043***	382 (122–644)	977 (523–1491)	***0*.*01***
**40–59**	42 (29–292)	605 (272–1434)	***0*.*0002***	490 (208–1187)	795 (383–1667)	***0*.*02***
**60–79**	173 (44–303)	607 (242–1555)		451 (121–609)	1558 (949–2733)	***0*.*01***
**Total**	**47 (37–252)**	**576 (225–1251)**	***<0*.*0001***	**469 (182–955)**	**812 (393–1906)**	***0*.*0002***

^a^Comparison of HIV^-^ and All HIV^+^ individuals (Mann Whitney U test).

^b^Comparison of immunoconcordant and immunodiscordant individuals. (Mann Whitney U test)

### Association between CMV IgG antibody levels, immunological variables and cell death

**Fig 2:**
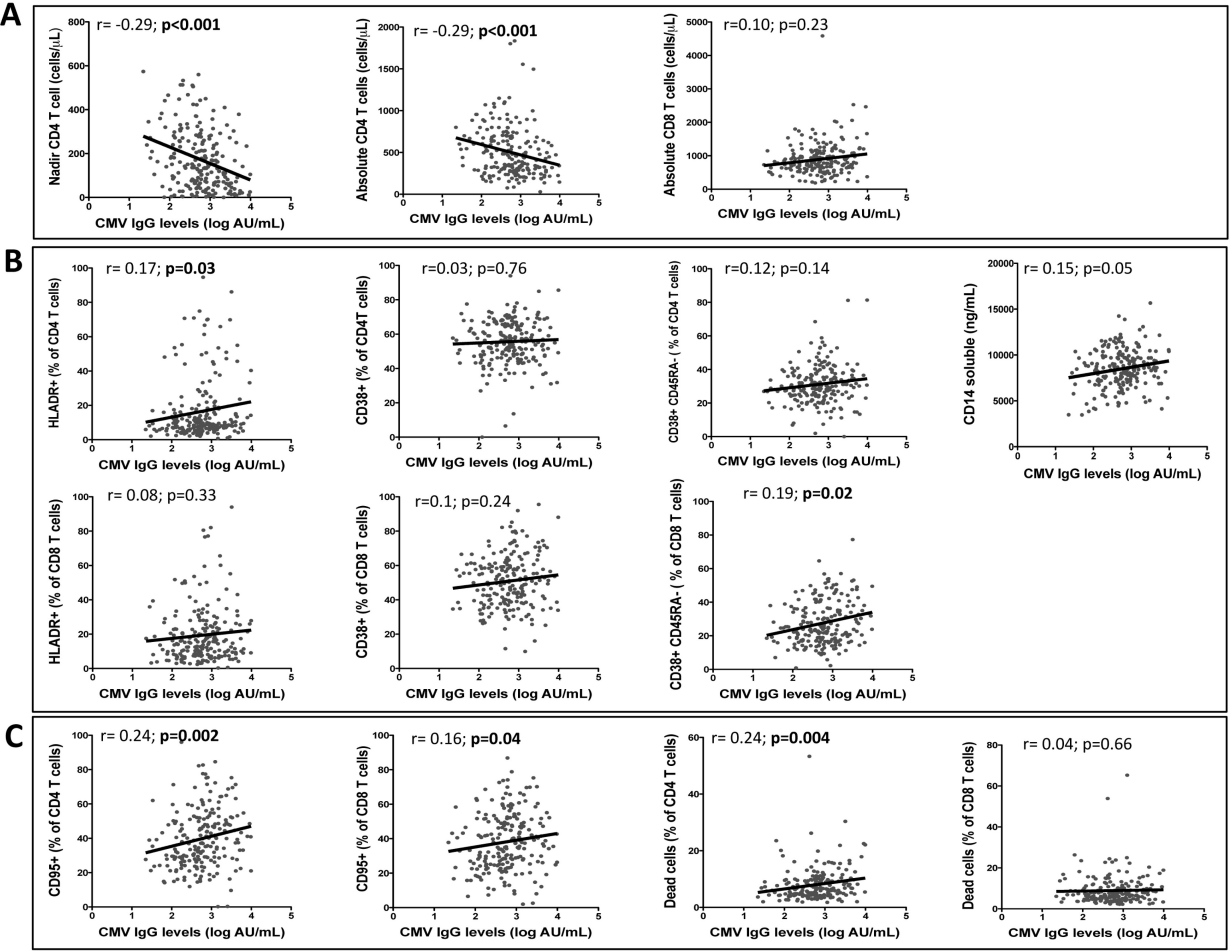
Correlations between CMV IgG antibody levels, immunological variables and T-cell destruction. A) Correlations between IgG levels and nadir CD4 T-cell counts, absolute CD4^+^ and CD8^+^ T-cell counts. B) Correlations between IgG levels and activation markers (measured as the percentage of HLADR and CD38) in CD4^+^ and CD8^+^ T-cells and in memory CD4^+^ and CD8^+^ T-cells (measured as the percentage of CD38) and the inflammatory marker sCD14. C) Correlations between IgG levels and the expression of the pro-apoptotic marker CD95 and total cell death in CD4^+^ and CD8^+^ T-cell populations. Total cell death was evaluated in 24-h *ex vivo* PBMC cultures. Linear correlation (Spearman) r and FDR-adjusted p-values are shown.

In HIV-infected individuals, no significant correlation was observed between CMV IgG levels and time since HIV diagnosis or time receiving antiretroviral treatment (Table 2 and S3 Fig). CMV IgG antibody levels were inversely correlated with nadir and absolute CD4^+^ T-cell counts (r = -0.29, p< 0.001; r = -0.29, p< 0.001, respectively) (Fig 2A and Table 1). No significant correlation was observed between CMV IgG levels and the percentage of naïve CD4^+^ T-cells (CD45RA^+^CD4^+^), whereas a trend was observed with naïve CD8^+^ CD45RA^+^ T-cells (r = -0.16, p = 0.05) (Table 2 and S3 Fig).

Regarding activation markers, CMV IgG levels were positively correlated with the expression of HLA-DR in CD4 T-cells (r = 0.17, p = 0.03, Fig 2B). In CD8^+^ T-cells, no significant correlation was observed between CMV IgG levels and CD38 or HLA-DR in the total population (Fig 2B), but a significant positive correlation with the frequency of activated memory (%CD38^+^CD45RA^–^) CD8^+^ T-cells (r = 0.19; p = 0.02) was observed (Fig 2B and Table 2). Furthermore, a trend was observed with plasma sCD14 levels (r = -0.15, p = 0.056) (Fig 2B and Table 2).

The CMV IgG antibody levels were not associated with T-cell production (CD31^+^ CD4^+^ or CD8^+^ cells), but they were highly correlated with the destruction of T-cells (Fig 2C, Table 2 and S3 Fig). In both CD4^+^ and CD8^+^ T-cells, CMV IgG levels were positively associated with the pro-apoptotic marker CD95 (r = 0.24, p = 0.002; r = 0.16, p = 0.04, respectively) (Fig 2C). Concordantly, a positive correlation was also observed with total cell death, necrosis and apoptosis of CD4^+^ T-cells evaluated in *ex vivo* culture of PBMCs. Conversely, CMV IgG levels were not associated with *ex vivo* CD8^+^ T-cell death or apoptosis (Fig 2C, Table 2 and S3 Fig).

### CMV IgM/ IgG avidity test

Plasma CMV IgM antibodies were measured in all study samples. Only 7 samples were positive, and all of them corresponded to HIV^+^ individuals, with an overall CMV IgM seropositivity of 3.1%. A higher presence, although not significant, was observed in the immunodiscordant group compared to the immunoconcordant group (4 of 95 samples and 3 of 133 samples, respectively). An IgG avidity assay was performed to determine whether the presence of IgM was due to a primary infection. In our cohort, all IgM^+^ samples exhibited high-avidity IgG (mean, 87.57%; SD±6.9), thus indicating that the infection was not primary and suggesting a reactivation process. However, a re-infection cannot be excluded.

To further explore the increased IgM presence in immunodiscordant individuals, a subgroup of subjects was selected from the total HIV^+^ group for whom we had three or more longitudinal plasma samples available over the course of HIV infection. A total of 812 samples were obtained from 58 immunoconcordant and 66 immunodiscordant subjects, and the presence of IgM was determined. The median number of samples by individual and the follow-up were higher in the immunoconcordant than in the immunodiscordant group (6 samples with 11.5 years of follow-up and 5 samples in 7.5 years, respectively) (Table 4). Among all the samples, 32 were IgM^+^, and all but one had high-avidity IgG antibodies (mean, 71.13%; SD±15.85). Remarkably, the distribution of the IgM^+^ samples was not homogeneous between the two HIV^+^ subgroups. Twenty-five of the IgM^+^/high-avidity IgG samples were from the immunodiscordant group in comparison with the 6 positive samples from immunoconcordant individuals (p = 0.0002) (Table 4). However, the number of individuals with IgM responses was not statically different between immunoconcordant and immunodiscordant individuals (Table 4, p = 0.41).

**Table 4:** Presence of anti-CMV IgM antibodies. IgM^+^/low-avidity IgG and IgM^+^/high-avidity IgG samples over the course of HIV infection.

	Immunoconcordant	Immunodiscordant	*p*- value
**Individuals, n tested**	58	66	
**Samples, n tested**	422	390	
**Samples/ Individuals, median (IQR)**	6 (4–10)	5 (4–7)	***0*.*02***^***a***^
**Follow-up, years median (IQR)**	11.5 (7–14)	8 (4–12)	***0*.*0003***^***a***^
**IgM**^**+**^**/low and high-avidity IgG samples, n (%)**	7 (1.65)	25 (6.4)	
**IgM**^**+**^**/high-avidity IgG samples, n**	6	25	***0*.*0002***^***b***^
**Individuals with IgM**^**+**^**/high-avidity IgG, n (%)**	5 (8.6)	9 (13.6)	*0*.*41*^*b*^
**IgM**^**+**^**/high-avidity IgG samples Rate, estimated**	0.16	0.45	***0*.*0222***^***c***^

^a^(Mann Whitney U test).

^b^(Fisher’s exact test).

^c^(Wald test from Poisson regression)

**Fig 3:**
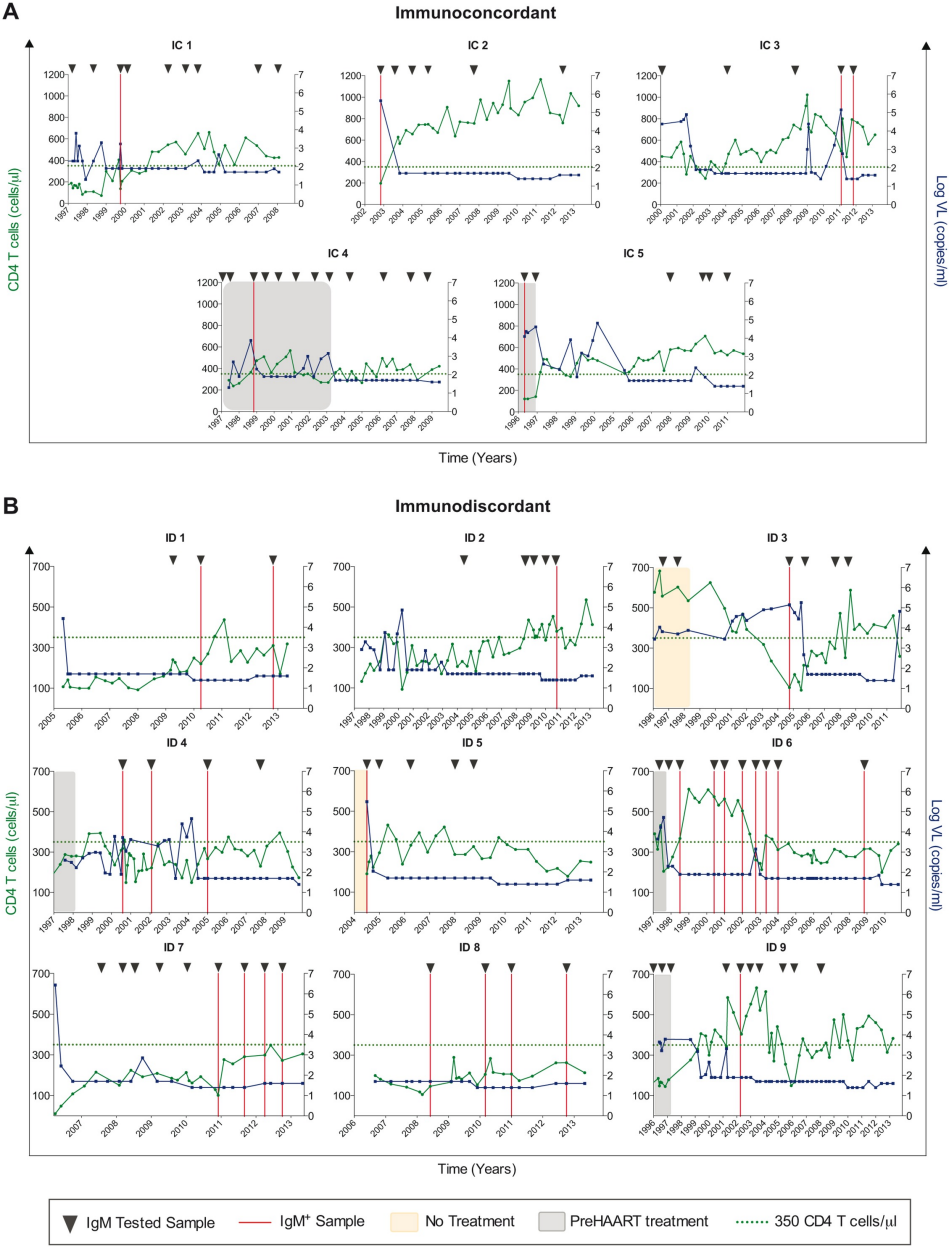
Pattern of CMV reactivation (IgM^+^/high-avidity IgG) in HIV-infected individuals. Evolution of HIV viral load (VL, blue lines) and CD4 T-cell counts (green lines) during HIV infection in immunoconcordant (A) and immunodiscordant (B) subjects is presented. Samples in which IgM antibodies were measured are indicated (closed arrowheads). Solid red lines represent IgM-positive/high-avidity IgG samples. Periods of no highly active antiretroviral therapy (pre-HAART period, grey areas) or without treatment (orange areas) are also depicted. Areas without color indicate that individuals were under HAART.

A more detailed analysis of subjects in whom the presence of IgM^+^/high-avidity IgG antibodies was detected showed that the pattern was different between immunoconcordant and immunodiscordant subjects (Fig 3A and 3B, respectively). The immunodiscordant group had a surprisingly high number of IgM+/high-avidity IgG samples during their follow-up. The difference was found to be statistically significant by fitting a Poisson regression model and adjusting the number of tested samples per individual (Table 4, p = 0.022). A portion of the immunodiscordant individuals had IgM antibodies present at most of the tested time points with a coverage of more than 9 years in one individual (ID 6, Fig 3B). Conversely, in immunoconcordant individuals, all but one presented IgM antibodies in only one of the tested samples (Fig 3A). The presence of IgM (high-avidity IgG) antibodies in immunoconcordant subjects was detected primarily in the first sample in the follow-up and was associated with peaks of HIV viremia and low CD4^+^ T-cell counts in the absence of HAART or during virological failure episodes. However, in immunodiscordant individuals, presence of IgM antibodies was not associated with HIV viral load, CD4^+^ T-cell count or lack of antiretroviral treatment (Fig 3B). In addition to IgM levels, the presence of CMV DNA in all plasma samples used in this study was analyzed using real-time PCR. All tested samples were negative.

### Association between CMV IgM or IgG antibody levels and CD4 T-cell destruction

**Fig 4:**
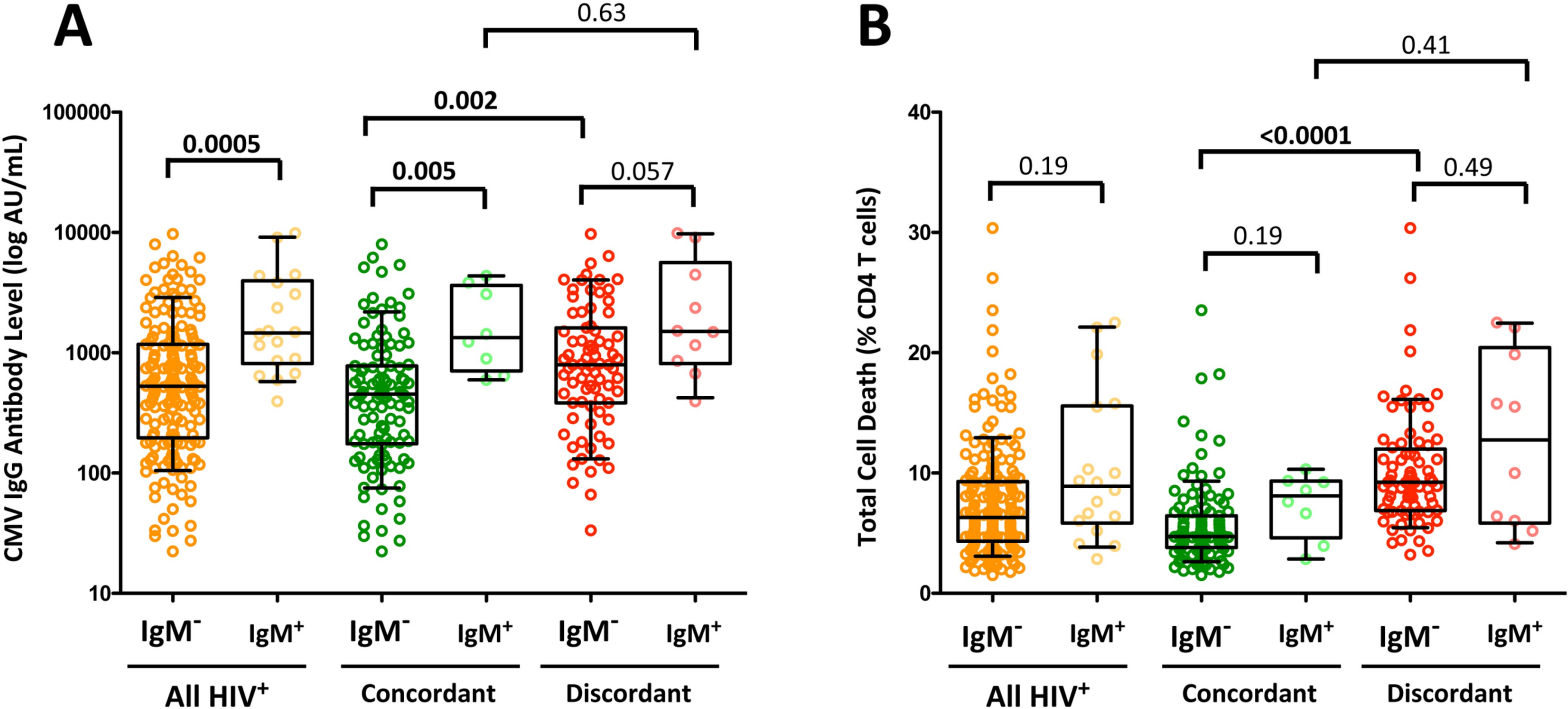
Association between CMV reactivation and CMV IgG antibody levels and T-cell death. Plasma CMV IgM antibody and IgG avidity was determined. Subjects with at least one IgM-positive/high-avidity IgG sample were classified as IgM-positive (IgM^+^) individuals (individuals with reactivations). Subjects in whom IgM was not detected are shown as IgM-negative (IgM^-^) individuals. The relationship between CMV reactivation and IgG levels (A) and total cell death (B) is shown. All HIV^+^ individuals (orange dots) and immunoconcordant (green dots) and immunodiscordant (red dots) subgroups are represented. The boxes represent the median and interquartile range of the values. Individual data of all subjects is displayed. The median values were compared using a non-parametric Mann-Whitney *U*-test. P-values have been corrected for multiple testing using Holm’s method.

The relationship between the presence of CMV IgM (with high-avidity IgG) antibodies and CMV IgG levels was evaluated in HIV^+^ individuals. Because IgM appears intermittently, participants who had at least one time point with detectable levels of IgM antibodies (n = 8, 6.5%, and n = 10, 11.2% in immunoconcordants and immunodiscordants, respectively) were compared to subjects with undetectable IgM levels at any time point analyzed during the study. The IgG antibody level exhibited a clear association with the presence of IgM antibodies with higher IgG levels in individuals in whom IgM antibodies were detected regardless of the group (Fig 4A). Total CD4^+^ or CD8^+^ T-cell death was not associated with the presence of IgM antibodies in any of the studied populations (Fig 4B and data not shown, respectively).

## Discussion

CMV IgG titers but not CMV serostatus were associated with CD4 immune recovery, and the highest values were observed in individuals with poor immune reconstitution. CMV IgG levels were also negatively associated with the percentage and the absolute CD4^+^ T-cell counts and with the nadir CD4^+^ T-cell count. Although our results were consistent with those of several previous studies [20–22,29], this association has not been observed by other authors [30,31]. Although potential factors related to such discrepancies may include geographic location and socioeconomic status of the studied populations, specific features of our study cohort may also be relevant, particularly the inclusion of individuals with a more compromised immunity and lower nadir and CD4^+^ T-cells numbers treated for longer times and, in some cases, in the pre-HAART era.

In addition, higher anti-CMV IgG levels were associated with an increased frequency of activated memory CD8^+^ T-cells (CD45RA^-^CD38^+^) and activated CD4^+^ T-cell populations (CD4^+^HLA-DR^+^) and a correlation with the inflammation marker sCD14. Although the associations were modest, these data were consistent with phenotypic changes previously described in CMV infection and its role in persistent immune activation in both HIV^+^ and HIV-uninfected populations [19,32,33]. sCD14, a marker of microbial translocation and monocyte activation, is associated with systemic immune activation [34,35] and is an independent predictor of non-AIDS defining disease and mortality in HIV-1-infected individuals [34,36,37]. Immunodiscordant subjects present higher levels of sCD14 than do immunoconcordant individuals [27,38]. Therefore, although the association between CMV IgG and sCD14 levels was modest, the higher levels of both parameters in immunodiscordant individuals may suggest that the reactivation of CMV contributes to the increased inflammation and mortality observed in these individuals. Recently, an association between CMV IgG levels and sCD14 has also been observed in a sub-Saharan Africa cohort of women [19].

It has been postulated that higher CMV IgG antibody levels represent more frequent or intense subclinical CMV reactivation from latency. In our study, CMV reactivation was evaluated by measuring DNAemia in plasma (the only available sample), and as might be expected by the low frequency of CMV in blood [30,39–41], none of the samples was positive. The IgM/IgG avidity test has been shown to be a reliable serologic indicator to distinguish primary infection from episodes of reactivation or re-infection [10,42]. Long-term IgM persistence after primary infection in certain individuals has been described. Twenty-five percent of patients with primary CMV infection still have detectable IgM 4 months after infection, and IgM occasionally persists for over a year [43–45]. However, the presence of IgM in our population was unlikely to be due to long-term IgM persistence, because IgM was detected in all the tested samples in certain analyzed individuals. These findings indicate persistence of more than 9 years that has not been reported to date. Using this test, IgM^+^/high-avidity IgG antibodies were found in 3.1% of the individuals in the cross-sectional study. Among the 124 subjects with longitudinal samples, 14 (11%) presented IgM antibodies. This prevalence was lower than that previously described by CMV DNA detection in other body fluids, which is in accordance with the compartmentalized replication of CMV primarily in the genital tract [40,46]. Interestingly, the number of IgM^+^/high-avidity IgG samples, likely episodes of reactivation although a re-infection process cannot be excluded, was not evenly distributed between HIV subgroups, and a different pattern was clearly observed, suggesting that the IgM presence could be triggered by different causes in those groups. In immunoconcordant individuals, IgM antibodies were found in isolated samples during the study period and was associated with high HIV viremia and/or low levels of CD4^+^ T-cells, which are both risk factors previously described for CMV reactivation [47]. In the immunodiscordant group, most individuals had more than one positive sample, and some of them had IgM^+^ samples at almost all the tested time points despite HIV suppression and CD4^+^ T-cell counts above 100 cells/μL, which is the cutoff level at which CMV reactivations are more frequent in HIV-infected individuals [47]. However, as CMV reactivation is induced by immunosuppression, the presence of IgM observed could be the result of the profound alterations in the memory CD4^+^ T-cell compartment described in immunodiscordant individuals [48]. Furthermore, the high rate of IgM^+^ samples may also be due to the persistently high levels of immune activation and inflammation displayed by those individuals [27]. Consistently, the initiation of CMV replication appears to be linked to the activation of the immune system, and high prevalence of CMV reactivation has been observed in conditions associated with increased levels of pro-inflammatory molecules [49]. Our study design does not allow us to establish the direction of causation in the relationship between CMV reactivation and inflammation; therefore, CMV reactivation could be the cause rather than the consequence of the immune activation and inflammation displayed by the immunodiscordant group.

The role of CMV infection in persistent immune activation and its contribution to poorer health have been extensively studied among HIV^+^ and HIV-uninfected populations [23,41,50,51], and the effect of CMV infection has been demonstrated by the significant reduction of activated CD8^+^ T-cells in blood after valganciclovir therapy [50]. We only found a trend in the increase of CD4^+^ and CD8^+^ T-cell activation likely due to the low number of samples in which we found reactivation (IgM antibodies). Cellular activation caused by CMV also contributes to HIV pathogenesis by depletion of T-cells via apoptosis-induced cell death [52]. According to these data, a correlation between IgG antibody levels and the frequencies of apoptosis-prone CD4^+^ and CD8^+^ T-cells and increased total cell death, apoptosis and necrosis in CD4^+^ T-cells from CMV IgM^+^ individuals was observed, suggesting that CMV infection may play an important role in CD4^+^ T-cell depletion in our HIV^+^ population.

Our study had several limitations. Unfortunately, the time of primary CMV infection was not available; thus, we cannot rule out the possibility that the higher level of CMV IgG in the immunodiscordant population could be due to a longer duration of CMV infection or an aberrant B-cell activation. A longer duration of CMV infection acquired before HIV infection may alter the balance between mature and naïve T-cells [32], skew the T-cell repertoire [53] and affect immune recovery. However, because this was an observational cross-sectional study, we could not establish a causal relationship between CMV IgG levels and immune recovery. We were also constrained by the small number of individuals with IgM^+^ samples in our cohort. However, the association between IgM seropositivity and the magnitude of IgG levels observed and previously reported [54] indicate that the IgM positivity is related to reactivation and has an impact on the systemic immune response. Conversely, a negative association between CMV seminal shedding and CMV IgG levels has been reported, although this association was found in a viremic-ART-naïve population with a high CD4 T-cell count [30]. However, the biological significance and the systemic impact of detectable CMV DNA in different compartments, the occurrence of CMV shedding, the frequency and the factors associated with the shedding in different body fluids are poorly understood [40,46,55,56].

Despite its limitations, the present study provides some insights regarding the connection between CMV infection, the intensity of the CMV humoral immune response and immune recovery. Specifically, this report describes an increased CMV humoral response in long-term treated HIV-infected individuals with poor immune recovery. This increase in the humoral response, along with its association with the nadir CD4^+^ T-cell count, CD4^+^ T-cell activation, sCD14 and cell death, suggest that CMV infection with likely persistent periods of subclinical reactivation could be a relevant driving force in inflammation and immune activation, and therefore, may impact the mortality observed in HIV^+^ individuals with poor CD4^+^ T-cell recovery. Further studies are needed to determine whether the persistent CMV replication could be targeted/prevented as a strategy to reduce the immune response intensity, persistent immune activation and mortality in those individuals at risk.

## Supporting information

S1 Fig Gating strategy followed to identify T-cell activation, production and destruction. CD4^+^ and CD8^+^ T-cells were gated from CD3^+^ lymphocytes. (A) Both T-cell populations were analyzed for the expression of the activation markers CD38 and HLA-DR and their percentage within the naïve (CD45RA^+^) and memory population (CD45RA^-^). (B) T-cell production and destruction was analyzed for the expression of the death receptor FAS (CD95), the activation marker HLA-DR, the exhaustion marker PD-1, the marker of recent thymic emigrant cells CD31 and the marker of T-cell maturation CD45RA. These markers were combined to determine the frequency of pro-apoptotic cells (HLA-DR^+^CD95^+^ and PD-1^+^CD95^+^), T-cell production (CD45RA^+^CD31^+^ cells) and the frequency of exhausted cells (HLA-DR^+^PD-1^+^).
(PDF)

S2 Fig Gating strategy for cell death analysis. Lymphocytes were gated according to morphological parameters. CD3 staining was used to identify T-cells that were further identified as CD4^+^ or CD8^+^ T-cells. Both populations were then analyzed for DiOC_6_ and PI staining allowing the identification of necrotic cells (PI^+^DiOC_6_^-^), apoptotic cells (PI^-^DiOC_6_^-^) and living cells (PI^-^DIOC_6_^+^).
(PDF)

S3 Fig Correlations between CMV IgG antibody levels and demographic and immunological variables. Correlations between IgG levels and demographic variables, activation levels and T-cell destruction are shown. Linear correlation (Spearman) r and FDR-adjusted p-values are displayed.
(PDF)
