# Supplementary figures and images for: Elevated humoral response to cytomegalovirus in HIV-infected individuals with poor CD4+ T-cell immune recovery
Source: PLoS One. 2017 Sep 21;12(9):e0184433. doi: 10.1371/journal.pone.0184433 (PMC5608209; doi:10.1371/journal.pone.0184433)

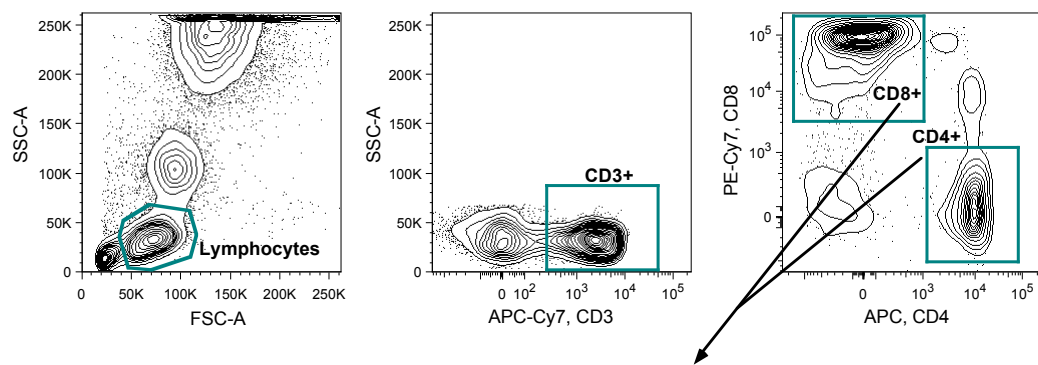

(A) Activation

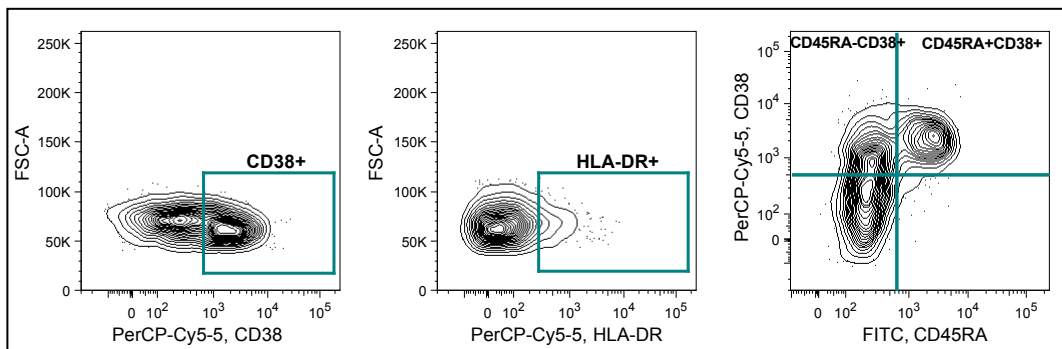

(B) T-cell production and destruction

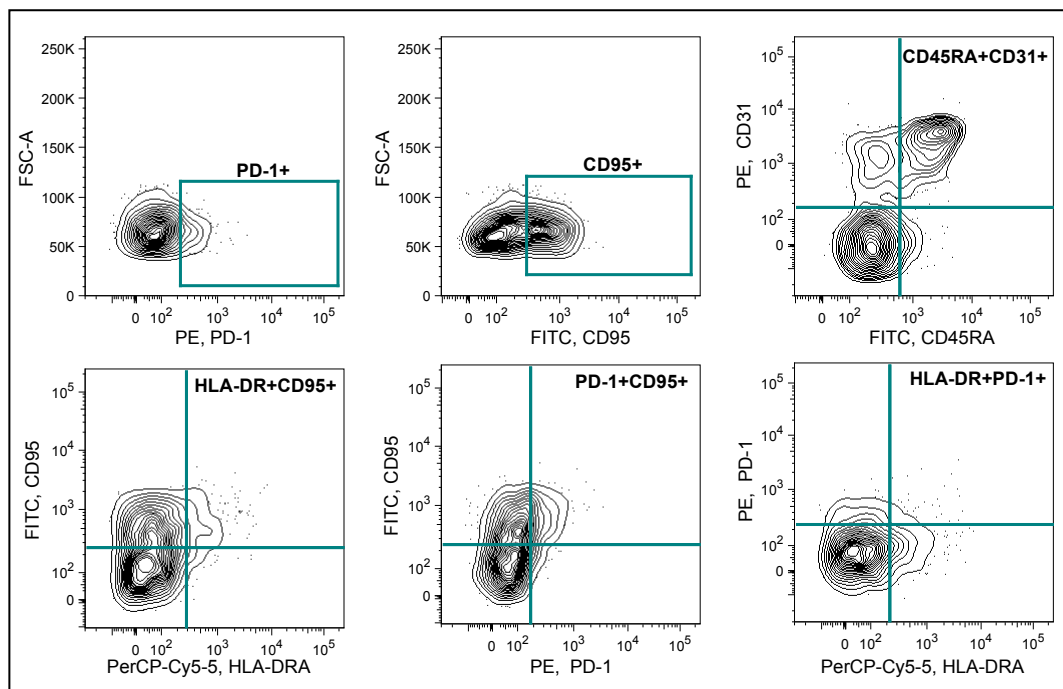

S1 Fig

Supplement: S1 Fig — CD4+ and CD8+ T-cells were gated from CD3+ lymphocytes. (A) Both T-cell populations were analyzed for the expression of the activation markers CD38 and HLA-DR and their percentage within the naïve (CD45RA+) and memory population (CD45RA-). (B) T-cell production and destruction was analyzed for the expression of the death receptor FAS (CD95), the activation marker HLA-DR, the exhaustion marker PD-1, the marker of recent thymic emigrant cells CD31 and the marker of T-cell maturation CD45RA. These markers were combined to determine the frequency of pro-apoptotic cells (HLA-DR+CD95+ and PD-1+CD95+), T-cell production (CD45RA+CD31+ cells) and the frequency of exhausted cells (HLA-DR+PD-1+). (PDF) [file pone.0184433.s001.pdf]

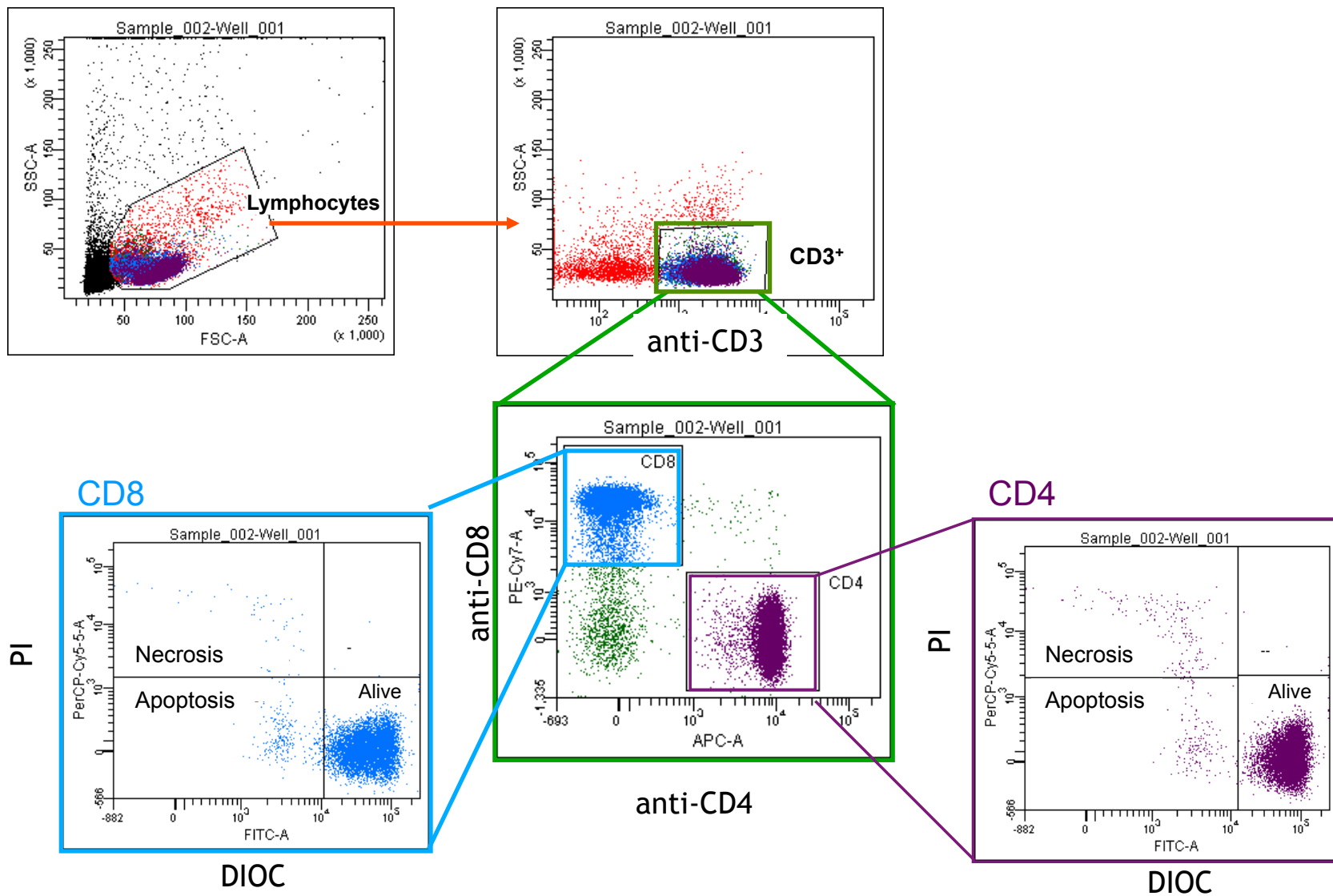

S2 Fig

Supplement: S2 Fig — Lymphocytes were gated according to morphological parameters. CD3 staining was used to identify T-cells that were further identified as CD4+ or CD8+ T-cells. Both populations were then analyzed for DiOC6 and PI staining allowing the identification of necrotic cells (PI+DiOC6-), apoptotic cells (PI-DiOC6-) and living cells (PI-DIOC6+). (PDF) [file pone.0184433.s002.pdf]
