# Supplementary material for: Elevated humoral response to cytomegalovirus in HIV-infected individuals with poor CD4+ T-cell immune recovery
Source: PLoS One. 2017 Sep 21;12(9):e0184433. doi: 10.1371/journal.pone.0184433 (PMC5608209; doi:10.1371/journal.pone.0184433)
Supplement: S3 Fig — Correlations between IgG levels and demographic variables, activation levels and T-cell destruction are shown. Linear correlation (Spearman) r and FDR-adjusted p-values are displayed. (PDF) [file pone.0184433.s003.pdf]

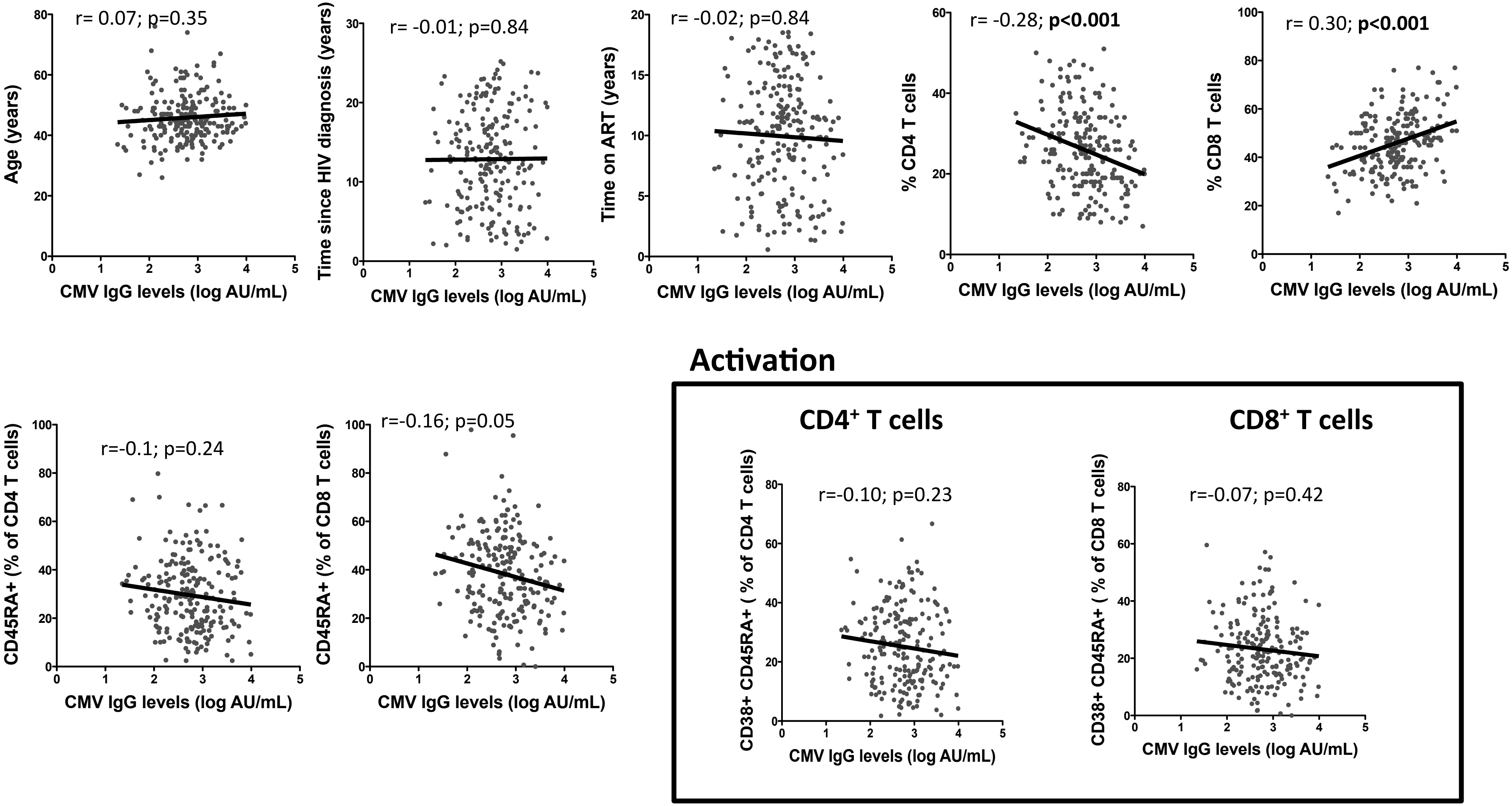
r= 0.07; p=0.35
Age (years)
CMV IgG levels (log AU/mL)
r= -0.01; p=0.84
Time since HIV diagnosis (years)
CMV IgG levels (log AU/mL)
r= -0.02; p=0.84
Time on ART (years)
CMV IgG levels (log AU/mL)
r= -0.28; **p<0.001**
% CD4 T cells
CMV IgG levels (log AU/mL)
r= 0.30; **p<0.001**
% CD8 T cells
CMV IgG levels (log AU/mL)
r=-0.1; p=0.24
CD45RA+ (% of CD4 T cells)
CMV IgG levels (log AU/mL)
r=-0.16; p=0.05
CD45RA+ (% of CD8 T cells)
CMV IgG levels (log AU/mL)
Activation
CD4⁺ T cells
r=-0.10; p=0.23
CD38+ CD45RA+ (% of CD4 T cells)
CMV IgG levels (log AU/mL)
CD8⁺ T cells
r=-0.07; p=0.42
CD38+ CD45RA+ (% of CD8 T cells)
CMV IgG levels (log AU/mL)

S3 Fig

T-Cell Production and Destruction. CD4^{+} T-cells

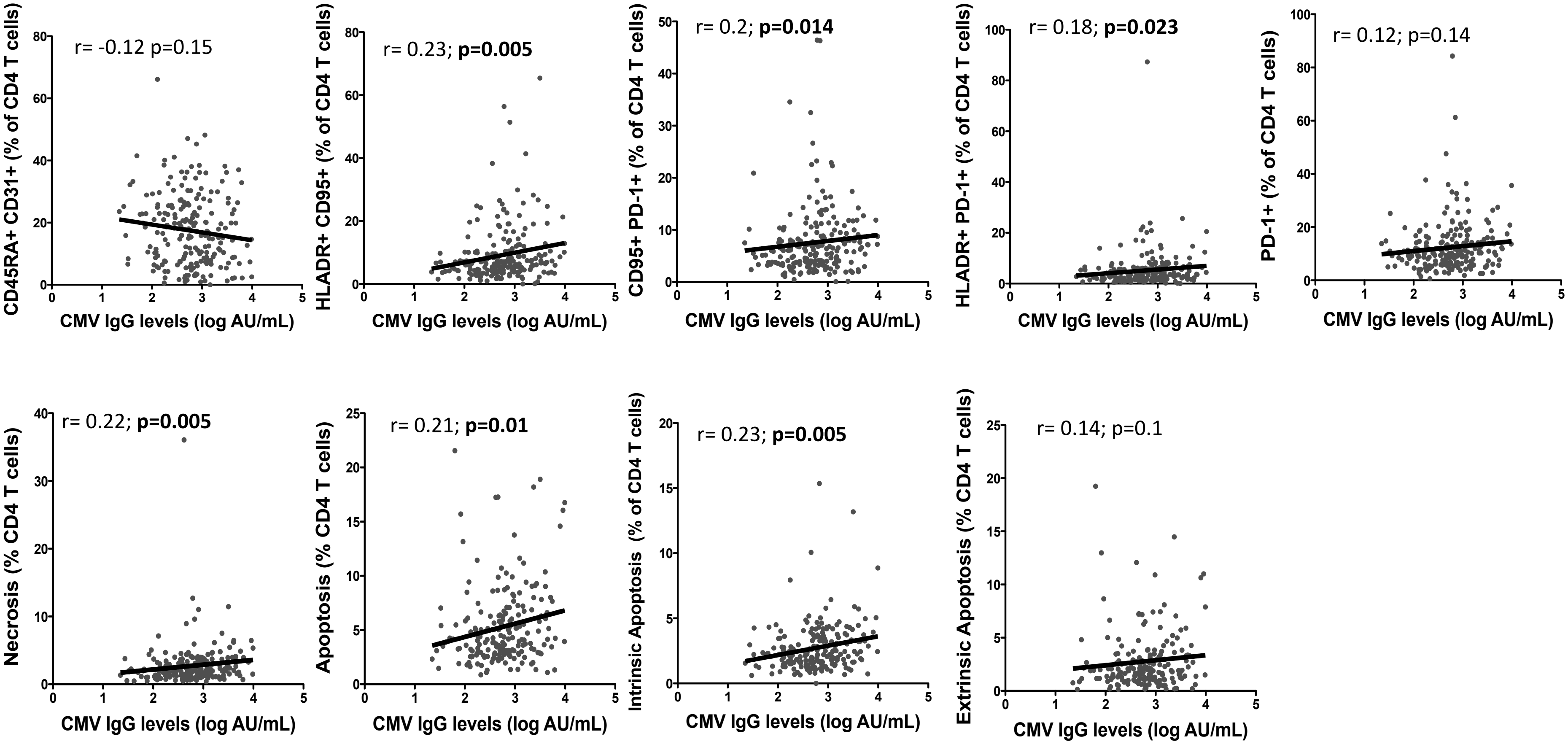

S3 Fig. Cont

T-Cell Production and Destruction. CD8^{+} T-cells

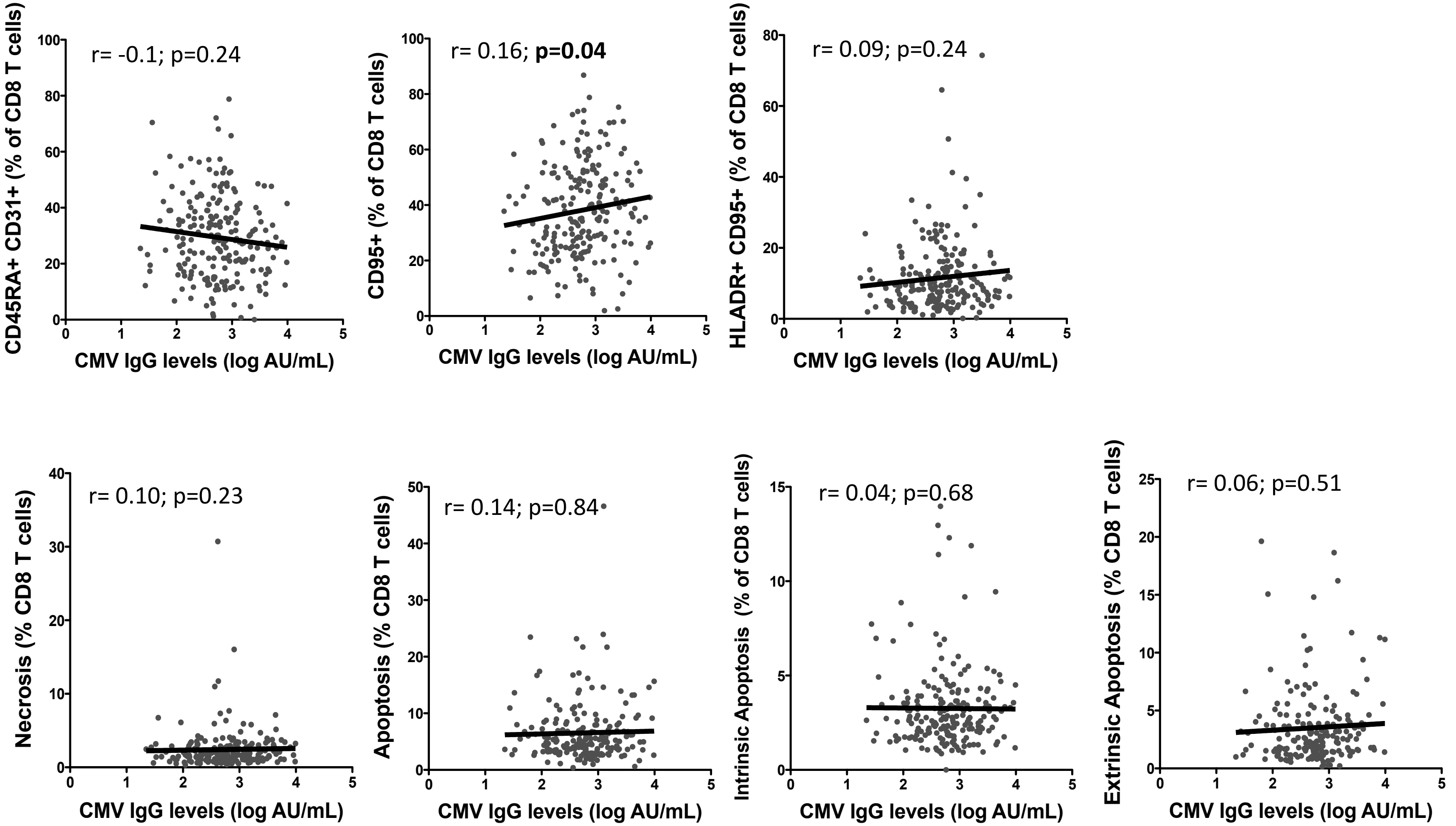

S3 Fig. Cont
